# The Burden of Noncommunicable Diseases in Ethiopia, 2000–2016: Analysis of Evidence from Global Burden of Disease Study 2016 and Global Health Estimates 2016

**DOI:** 10.1155/2020/3679528

**Published:** 2020-02-19

**Authors:** Tadele Girum, Dereje Mesfin, Jemal Bedewi, Misgun Shewangizaw

**Affiliations:** ^1^Department of Public Health, College of Medicine and Health Sciences, Wolkite University, Wolkite, Ethiopia; ^2^Department of Public Health, College of Medicine and Health Sciences, Arba Minch University, Arba Minch, Ethiopia

## Abstract

**Background:**

The continuing rise in the burden of noncommunicable diseases (NCDs) is a key global health agendum due to the fact that NCDs cause more deaths than all other causes combined together. Although measuring the burden of NCD is very important to improve the existing health care systems and to monitor the progress of the program, a comprehensive estimate is lacking in Ethiopia. Hence, we aimed to systematically analyze the existing evidence to bring a solution.

**Methods:**

The research used data from the Global Burden of Disease Study (GBD 2016) and Global Health Estimates 2016 that originally collected the information through vital registration, verbal autopsy, surveys, reports, and modeling.

**Results:**

In 2016, NCD caused an estimated 274998.8 (95% CI: 211290.2–362882.1) deaths among all ages and both genders with a crude death rate of 268.5/100000 and age-standardized death rate (ASDR) of 554.7/100000 population. It contributed to 39.3% of the total death, 53% of ASDR, and 34% of DALYs. The number of deaths and DALYs from NCD has increased by 38% and 31.5%, respectively, whereas CDR and ASDR from NCD have declined by 10.3% and 12.5%, respectively. Cardiovascular diseases, malignant neoplasms, digestive diseases, respiratory diseases, diabetes mellitus, and neurological conditions were the leading level 2 causes of ASDR due to NCD, while ischemic heart disease, stroke, other circulatory diseases, cirrhosis of the liver, and COPD were the top 5 causes of ASDR from NCD at level 3 causes. *Conclusion and Recommendation*. The burden of NCD was remarkably increased between 2000 and 2016. It carries the highest burden of ASDR. Cardiovascular diseases and malignant neoplasms were the two most common causes of mortality and DALYs. Therefore, the existing disease prevention strategies should be strengthened by incorporating strategies addressing noncommunicable diseases.

## 1. Introduction

Noncommunicable diseases (NCDs) are diseases or conditions which are usually of chronic nature, with slow onset, lengthy progression for which there are no known causative agents, and generally are nontransmittable from one person to another [1, 2].

The continuing rise in the burden of NCDs is a key global health agendum due to the fact that NCDs cause more deaths than all other causes combined together [3]. According to recent estimates of WHO, globally more than 41 million (71%) deaths were from NCDs, and nearly 78% of all NCD deaths and 85% premature NCD deaths occur in low- and middle-income countries which are higher than those in developed countries [4].

The highest burden of NCDs can be attributed to chronic diseases which once known to be the ailments of affluence such as cardiovascular diseases, cancers, chronic respiratory illnesses, and diabetes which, respectively, account for 44%, 9%, 9%, and 4% of all NCD-related deaths [4]. The harmful use of alcohol, tobacco use, dietary behaviors, and physical inactivity are among behavioral risk factors for NCDs, and raised blood pressure, raised blood glucose, and obesity are among established metabolic risk factors for NCDs [5].

It is projected that the burden of NCDs will continue to rise in low-income countries such that by the coming 2030, NCDs will cause eight times more mortality in low- and middle-income countries (LMICs) compared to the developed ones [6] which will make things worst considering the fact that developing countries are still struggling with infectious diseases, civil war, poverty, road traffic accidents, and workplace injuries which will make them crippled by triple burden of disease [7]. Furthermore, NCDs affect productive age groups, and adults in low- and lower-middle-income countries had double risk of dying from an NCD compared with adults in high-income countries creating a vicious cycle of poverty and ill-health in LMICs [7, 8].

Even though there is a lack of reliable and accurate data on the prevalence of NCDs in sub-Saharan Africa, NCDs are expected to exceed infectious diseases as major causes of morbidity and mortality by the year 2035 [9]. Ethiopia is one of the fastest growing economies undergoing a rapid economic transformation in the region [10]. The burden of NCDs in Ethiopia is similar to that of other developing nations, which is estimated to account for 39% of all deaths with the leading causes being cardiovascular diseases and cancer [4, 10].

Though the Ethiopian Ministry of Health has adopted the global declaration to strengthen global and national responses to prevent and control NCDs [11] and considered them in the health sector transformation Plan (HSTP) of the country as one of the prioritized disease control areas [12], the country's capability to achieve those targets is questionable considering that only half of the health service facilities are ready to provide general NCD services and minimum government expenditure on the area whereby 68% of NCDI services are financed by out-of-pocket (OOP) expenditures from households [10].

Despite major progresses have been made to reduce the burden of infectious diseases and mortality from maternal and child health, the burden of noncommunicable diseases estimated to be higher. However, the actual causes of such higher mortality and DALYs from NCD were not well understood. Therefore, this study was aimed at measuring the burden of NCD disease and identifying causes of mortality in Ethiopia between 2000 and 2016 by using evidence from the Global Burden of Disease Study 2016 (https://vizhub.healthdata.org/gbd-compare/) and Global Health Estimates 2016 (http://www.who.int/healthinfo/global_health_estimates/en/), which will contribute to the improvement of the health status of the population.

## 2. Methods and Material

### 2.1. Study Area and Source of Data

The burden of disease and cause of mortality are periodically estimated by the World Health Organization and IME since 1990. This research is based on a systematic analysis of the global burden estimates for Ethiopia, which is the second populous country next to Nigeria with a population estimated at 102 million in 2017 of which 83.86 percent live in rural areas [13]. The main sources of data for this research are the WHO Global Health Estimates data base (http://www.who.int/healthinfo/global_health_estimates/en/) in which estimates are available for years 2000, 2005, 2010, 2015, and 2016 for member states and for selected regional groupings of countries, areas, and territories and Institute for Health Metrics and Evaluation (IHME) (Global Burden of Disease Study 2016) data base (http://vizhub.healthdata.org/gbd-compare/). The organization collects, organizes, and estimates disease burden in collaboration with other United Nations agencies such as UNICEF, UNAIDS, and UNFPA and academic and research institutions. The estimation is particularly based on vital registries, surveys, researches, and model estimations.

### 2.2. Operational Definition

Disability is used broadly in disease burden analyses to refer to departures from good or ideal health in any of the important domains of health.

Disability-adjusted life year (DALY) is a summary measure which combines time lost through premature death and time lived in states of less than optimal health, loosely referred to as “disability.”

### 2.3. Statistical Analysis and Interpretation

This study analyzed on the burden of noncommunicable diseases in Ethiopia from the general measurement of disease burden including group I (communicable diseases, maternal and child problems, and nutritional problems), group II (NCD), and group III (injuries). The GBD study and GHE approaches to estimate all-cause mortality and cause-specific mortality rates by age, sex, and year have been described elsewhere [14, 15]. Causes of death by age, sex, and year for all causes were measured mainly using cause of death ensemble modeling (CODEm) that models different statistics and estimate outcomes on the basis of the performance of fitted models [16]. DALY was measured by summing years of life lost (YLL) due to premature mortality and years lived with disability (YLD), a measure of nonfatal health loss, in a single metric. YLL were estimated using standard GBD methods whereby each death is multiplied by the normative standard life expectancy at each age. YLD were estimated using sequelae prevalence and disability weights derived from population-based surveys. For most sequelae, the GBD 2016 study used a Bayesian meta-regression method, DisMod-MR 2.1, designed to address key limitations in descriptive epidemiological data, including missing data, inconsistency, and large methodological variation between data sources [14, 15].

## 3. Results

Noncommunicable diseases caused an estimated 274998.8 (95% CI: 211290.2–362882.1) deaths among all ages and both gender groups in 2016. Death from NCD also has contributed to 39.3% of the total death 700108.8 from all causes estimated to have occurred in the year 2016. Even though the total death from all causes was estimated to have declined by 28.7% between 2000 and 2016, the number of deaths from NCD has increased by 38% in the same period. By the same year, the crude death rate related to NCD was estimated to be 268.5/100000 and age-standardized death rate was 554.7/100000 population. Both declined from the 2000's record only by 10.3% and 12.5%, respectively (Tables 1 and 2).

Among the level 2 causes of NCD, cardiovascular diseases contributed to the highest number of deaths 109499.7 (95% CI: 80701.5–150979.3) followed by malignant neoplasms that caused 50913.5 (95% CI: 36092.1–73018.8) deaths in the year 2016. Also, diabetes mellitus, mental and substance use disorder, neurological conditions, respiratory diseases, and digestive diseases caused 13027.9, 1926.9, 12700.2, 15459.8, and 36868.2 deaths, respectively. The crude death rate resulted from the two most common causes of NCD: cardiovascular diseases and malignant neoplasms were 106.9/100000 and 49.7/100000 population, respectively (Table 1).

Of the level 3 causes, ischemic heart disease, stroke, other circulatory diseases, cirrhosis of the liver, diabetes mellitus, other malignant neoplasms, and COPD caused the highest number of deaths with 47711.6, 32859.5, 16767.0, 16579.5, 13027.9, 10916.3, and 10347 deaths, respectively. The number of deaths due to Alzheimer disease and dementias, ovary cancer, lymphomas and myeloma, ischemic heart disease, other circulatory diseases, other malignant neoplasms, cardiomyopathy and myocarditis, and hypertensive heart disease has increased by more than 50% between 2000 and 2016. Particularly, the number of deaths from Parkinson disease, Alzheimer disease and dementias, drug use disorders, and ovary cancer increased by more than 100% (Table 1).

NCD was the highest contributor of age-standardized death rate in the year 2016. It contributed to 53% of ASDR. Among the level 2 causes, cardiovascular diseases and malignant neoplasms caused the highest ASDR with a rate of 252.9/100000 and 93.5/100000 population, respectively, in the year 2016, while digestive diseases, respiratory diseases, diabetes mellitus, and neurological conditions caused a death rate of 69.5/100000, 34.0/100000, 29.1/100000, and 30.3/100000 population, respectively. Although most of ASDR from level 2 causes has declined, the number of deaths from other neoplasms, neurological conditions, and skin diseases was increased between 2000 and 2016 (Table 2).

The major cause of ASDR at level 3 category was ischemic heart disease with a death rate of 112.4/100000 population in 2016, with decreasing orders: stroke (74.9/100000), other circulatory diseases (38.7/100000), cirrhosis of the liver (30.9/100000), and COPD (24.6/100000) ranked as the top 5 causes of ASDR from NCD. Mortality from these common causes of ASDR slightly declined in the last few decades; however, mortality from other causes of NCD such as ovary cancer, drug use disorders, other endocrine and immune disorders, Alzheimer disease and other dementias, Parkinson disease, and most other forms of cancer increased by more than 10% between 2000 and 2016 (Table 2).

Disability-adjusted life years (DALYs) lost due to NCD in all ages were 15849800 in 2016, which increased by 31.5% from the 2000's record of 12053500 DALYs. Cardiovascular diseases accounted for 19% (3009900/15849800) of the national NCD-related DALYs, with malignant neoplasms leading to 12.7% (2012500/15849800) and mental and substance use disorders to 13.2% (2089700/15849800). Since 2000, DALYs due to all level 2 causes have been increased substantially except for endocrine, blood, and immune disorders and sudden infant death syndrome (Table 3).

DALY from level 2 causes of NCD diseases especially malignant neoplasms, other neoplasms, diabetes mellitus, mental and substance use disorders, neurological conditions, skin diseases, and musculoskeletal diseases increased by 45-109%. Similarly, at individual-level causes, DALY from ovary cancer, eating disorders, and Alzheimer disease and other dementias increased by more than 100%. Also, DALYs from most of malignancies, cardiovascular diseases, mental problems, and substance use disorders and neurologic disorders increased by more than 50% (Table 3).

## 4. Discussion

This study assessed the burden of NCD among all ages in Ethiopia from 2000 to 2016 evidenced from the Global Burden of Disease Study measurement and Global Health Estimates of 2016 reported in 2018. The burden was measured in terms of mortality and disability-adjusted life years. Accordingly, the study evidenced that the number of deaths from NCD and DALYs lost due to NCD was highly increased between 2000 and 2016, while crude death rate and ASDR were slightly decreased in the same period. In lower-income countries, the increase in the relative burden from noncommunicable disease and the decrease in communicable disease burden are occurring more rapidly than in high-income countries [17, 18].

An estimated 274998.8 (95% CI: 211290.2–362882.1) deaths among all ages and both gender groups were reported due to NCD in 2016. Thus, death from NCD has contributed to 39.3% of the total number of deaths from all causes estimated to have occurred in the year 2016. In the same year, the crude death rate related to NCD was estimated to be 268.5/100000 and age-standardized death rate was 554.7/100000 population. The number of mortality due to NCD increased by 38% between 2000 and 2016. The population growth partly contributed to the increased number of NCD. Also, the existing epidemiological shift observed in most developing countries has the major role [17–19].

Interventions designed to address noncommunicable diseases were poorly implemented in developing countries. Until recently, the Ethiopian government has not designed a preventive strategy to overcome the burden of noncommunicable diseases. Also, global interventions that were designed yet and interventions taken at the millennium development goal and sustainable development programs were particularly targeted on infectious diseases and maternal and child health and nutritional problems, and less emphasis was given for NCD; this may have contributed to the existing higher burden of NCD [18–20].

Cardiovascular diseases and malignant neoplasms were the two major causes of NCD-related mortality. In our case, cardiovascular diseases contributed to 109499.7 (95% CI: 80701.5–150979.3) deaths, and malignant neoplasms caused 50913.5 (95% CI: 36092.1–73018.8) deaths. It is in line with the global epidemiology, where cardiovascular and other noncommunicable diseases carry the highest share of mortality [17, 20]. According to WHO estimate, cardiovascular diseases contribute to 9% of the total death and 30% of deaths were due to noncommunicable diseases in Ethiopia in 2012. Additionally, a study conducted in Addis Ababa indicated that about 51% of deaths were due to NCDs and 24% of deaths were due to cardiovascular diseases [17].

Similarly, the commonest NCDs in the country such as diabetes mellitus, mental and substance use disorders, neurological conditions, respiratory diseases, and digestive diseases significantly contribute to the existing higher burden of NCD-related mortality and morbidity. At the individual disease level (the level 3 causes), ischemic heart disease, stroke, other circulatory diseases, cirrhosis of the liver, diabetes mellitus, other malignant neoplasms, and COPD were found to cause the highest number of deaths [18]. As evidenced from previous researches, cancer, chronic obstructive pulmonary diseases, and diabetes mellitus were contributing to 6%, 3%, and 1%, respectively, of the total deaths in Ethiopia [1, 21].

Currently, the epidemiology of most noncommunicable disease was highly increasing from time to time. In our study, the number of deaths due to Alzheimer disease and dementias, ovary cancer, lymphomas and myeloma, ischemic heart disease, other circulatory diseases, other malignant neoplasms, cardiomyopathy and myocarditis, and hypertensive heart disease has increased by more than 50% between 2000 and 2016. Particularly, the number of deaths from Parkinson disease, Alzheimer disease and dementias, drug use disorders, and ovary cancer increased by more than 100%. Although the epidemiology of these chronic diseases was higher in the global epidemiology, their burden is remarkably higher in developing countries like Ethiopia [17–20].

NCD contributed to 53% of ASDR and 34% of total DALYs. It is slightly lower than the global average where around 60-70% of ASDR was due to NCD. But rapid change was observed from 30% contribution in 2012 to 53% contribution in 2016 that can evidence the epidemiological shift observed in Ethiopia. Also, a previous study reported that noncommunicable diseases were the leading causes of age-standardized mortality rates whereas communicable and maternal and child health problems and nutritional problems were the leading causes of premature mortality in Ethiopia [19, 21].

Cardiovascular diseases, malignant neoplasms, digestive diseases, respiratory diseases, diabetes mellitus, and neurological conditions were the leading level 2 causes of ASDR due to NCD, while ischemic heart disease, stroke, other circulatory diseases, cirrhosis of the liver, and COPD were the top 5 causes of ASDR from NCD at level 3 causes. The previous studies also reported that cardiovascular and malignant conditions are increasing [19, 21]. It is also in line with the global report that ischemic heart disease is the leading cause of age-standardized death [18, 20].

Disability-adjusted life years (DALYs) lost due to NCD in all ages were 34% which increased by 31.5% from the 2000's report. Specially, malignant neoplasms, other neoplasms, diabetes mellitus, mental and substance use disorders, neurological conditions, skin diseases, and musculoskeletal diseases increased by 45-109%. It is also in line with the global trend of noncommunicable diseases, where the burden is highly increasing [18, 20]. Globally, group II causes (NCD) accounted for 61.4% of the global DALY in 2017. Even though DALYs lost due to NCD declined by 37% between 1990 and 2016, the burden was not reduced remarkably as it was in communicable diseases and maternal and child health problems [16–18].

The findings of this study might suffer from the fact that it is the secondary data based on records; the reliability of the recorded data could not be ascertained, and potential bias associated with estimation is there. Some methodological problems may have been encountered in this research. Most of the data were originally estimated from model predictions, and the data source for the model was either reports of vital registration or sample survey that could again affect the reliability of the data. Moreover, the forecasted values from the trend may change through time due to change in intervention programs; this in turn affects the reliability of the estimate.

## 5. Conclusion and Recommendation

Noncommunicable diseases contributed to 39.3% of the total death, 53% of ASDR, and 34% of DALYs from all causes. The number of deaths and DALYs from NCD has increased by 38% and 31.5%, respectively, whereas CDR and ASDR from NCD have declined by 10.3% and 12.5%, respectively. Cardiovascular diseases, malignant neoplasms, digestive diseases, respiratory diseases, diabetes mellitus, and neurological conditions were the leading level 2 causes of ASDR due to NCD, while ischemic heart disease, stroke, other circulatory diseases, cirrhosis of the liver, and COPD were the top 5 causes of ASDR from NCD at level 3 causes.

Therefore, the existing disease prevention strategies should be strengthened by incorporating strategies addressing noncommunicable diseases, and a particular emphasis should be given to high-burden noncommunicable disease.

## Figures and Tables

**Table 1:** Number and cause of deaths from NCD for both sexes and all age groups from 2000-2016.

Cause of death	Number of deaths in 2000			Number of deaths in 2016			%Change
	Value	95% LL	95% UL	Value	95% LL	95% UL	
All causes	**981955.9**	**867049.4**	**1107745.6**	**700108.8**	**588955.7**	**831398.4**	−**28.7**
Noncommunicable diseases	**199299.9**	**158916.3**	**248625.1**	**274998.8**	**211290.2**	**362882.1**	**38.0**
Malignant neoplasms	34534.5	25677.2	46261.7	50913.5	36092.1	73018.8	47.4
Mouth and oropharynx cancers	870.6	542.0	1344.9	1373.1	798.5	2275.8	57.7
Oesophagus cancer	1453.3	929.2	2191.6	1651.1	972.8	2706.9	13.6
Stomach cancer	1490.7	949.5	2255.6	1511.1	883.1	2494.1	1.4
Colon and rectum cancers	2370.5	1538.3	3525.7	3475.1	2116.5	5533.4	46.6
Liver cancer	654.8	402.6	1022.6	1102.1	633.6	1844.5	68.3
Pancreas cancer	297.2	176.0	479.5	552.6	305.8	954.4	85.9
Trachea and lung cancers	1005.7	630.0	1545.0	1681.9	988.4	2762.8	67.2
Melanoma and skin cancers	139.3	79.4	231.9	231.4	121.6	416.1	66.1
Breast cancer	5184.9	3547.7	7332.7	7653.6	4961.7	11508.1	47.6
Cervix uteri cancer	4427.3	3014.5	6290.7	5013.5	3197.1	7655.4	13.2
Corpus uteri cancer	183.4	109.7	293.5	246.1	135.2	427.5	34.2
Ovary cancer	1200.2	779.2	1784.4	2511.2	1554.6	3940.3	109.2
Prostate cancer	702.9	446.6	1065.9	1384.4	833.8	2226.2	97.0
Testicular cancer	82.6	47.4	136.7	77.1	39.3	141.8	−6.6
Kidney cancer	741.4	459.0	1151.1	1282.4	743.9	2130.1	73.0
Bladder cancer	617.5	379.5	965.0	937.4	536.0	1576.2	51.8
Brain and NS system cancers	264.2	156.0	427.2	508.9	281.4	879.3	92.7
Gallbladder and biliary cancer	445.2	272.1	699.4	587.3	330.2	1001.6	31.9
Larynx cancer	137.4	78.7	228.0	180.5	94.3	325.9	31.3
Thyroid cancer	1045.1	658.0	1598.4	1390.0	810.6	2298.5	33.0
Mesothelioma	68.2	37.3	117.2	74.1	35.8	141.5	8.7
Lymphomas and myeloma	1897.3	1220.6	2844.6	3098.6	1877.3	4957.1	63.3
Leukaemia	2317.4	1502.7	3449.4	3473.7	2115.3	5531.9	49.9
Other malignant neoplasms	6937.3	4684.3	9937.3	10916.3	6985.9	16614.0	57.4
Other neoplasms	1119.1	645.1	1845.0	2537.3	1391.3	4386.9	126.7
Diabetes mellitus	8058.9	4956.3	12447.5	13027.9	7589.9	21245.6	61.7
Endocrine, blood, and immune disorders	4775.1	2888.9	7500.9	4144.7	2316.2	7038.5	−13.2
Thalassaemias	1237.1	523.4	2569.5	731.5	288.0	1592.9	−40.9
Sickle cell disorders and trait	1054.3	443.4	2200.5	731.4	288.0	1592.6	−30.6
Other haemoglobinopathies	1838.7	789.2	3773.0	1655.2	673.3	3514.0	−10.0
Other immune disorders	645.0	266.1	1367.3	1026.6	409.9	2211.5	59.2
Mental and substance use disorders	1249.6	752.0	2015.7	1926.9	1058.5	3431.3	54.2
Alcohol use disorders	957.4	602.6	1464.7	1309.1	764.7	2162.1	36.7
Drug use disorders	290.5	173.9	464.5	614.6	344.9	1049.4	111.6
Eating disorders	1.7	0.7	3.7	3.2	1.2	7.3	81.8
Neurological conditions	7281.4	4928.1	10406.8	12700.2	8177.1	19218.8	74.4
Alzheimer and dementias	3615.3	2167.6	5731.4	8316.4	4769.8	13772.9	130.0
Parkinson disease	221.6	129.8	361.0	544.4	301.4	939.7	145.6
Epilepsy	3136.4	2065.4	4601.8	3342.2	2040.4	5311.5	6.6
Multiple sclerosis	23.7	12.1	43.0	40.8	18.8	80.4	71.8
Other neurological conditions	284.3	168.0	459.7	456.4	249.9	794.8	60.5
Cardiovascular diseases	76252.4	58615.4	98787.6	109499.7	80701.5	150979.3	43.6
Rheumatic heart disease	2018.5	1301.7	3019.7	1449.6	845.7	2396.2	-28.2
Hypertensive heart disease	5106.9	3097.0	8002.7	7754.3	4436.5	12872.8	51.8
Ischemic heart disease	30060.2	21287.7	41090.7	47711.6	32277.9	68837.1	58.7
Stroke	26536.2	18716.1	36420.1	32859.5	21936.9	48029.7	23.8
Cardiomyopathy, endocarditis	1919.4	1236.3	2874.6	2957.8	1790.3	4736.0	54.1
Other circulatory diseases	10611.3	7271.7	14983.7	16767.0	10915.1	25109.7	58.0
Respiratory diseases	12300.6	8470.8	17286.0	15459.8	10032.7	23221.2	25.7
COPD	7323.6	4958.6	10463.3	10347.0	6609.1	15775.4	41.3
Asthma	4538.7	2740.8	7142.8	4484.8	2514.2	7593.1	-1.2
Other respiratory diseases	438.3	179.1	936.5	628.0	246.4	1371.4	43.3
Digestive diseases	29438.5	20849.5	40238.0	36868.2	24736.9	53627.7	25.2
Peptic ulcer disease	4245.3	2558.3	6695.3	4198.9	2347.6	7127.4	-1.1
Cirrhosis of the liver	11958.2	7444.9	18237.6	16579.5	9745.3	26800.5	38.6
Appendicitis	1364.8	792.3	2234.9	1206.1	641.7	2144.5	-11.6
Gastritis and duodenitis	731.4	416.0	1221.7	1125.0	598.2	2001.5	53.8
Paralytic ileus and IO	5755.1	3508.1	8971.4	7449.6	4262.5	12366.9	29.4
Inflammatory bowel disease	479.5	268.0	814.5	791.3	413.4	1430.1	65.0
Gallbladder and biliary disease	767.7	436.8	1281.9	1177.7	626.3	2094.7	53.4
Pancreatitis	486.8	273.6	823.0	683.7	356.6	1237.9	40.4
Other digestive diseases	3649.8	2196.4	5765.0	3656.5	2038.4	6224.4	0.2
Genitourinary diseases	6717.0	4112.3	10424.0	9329.6	5377.4	15376.7	38.9
Kidney diseases	4549.3	2748.8	7155.4	6426.2	3650.7	10741.6	41.3
Urolithiasis	34.1	17.4	63.1	66.9	31.6	132.0	96.2
Other urinary diseases	1917.6	1140.4	3065.9	2615.9	1455.3	4462.7	36.4
Gynecological diseases	216.0	120.3	368.4	220.6	112.3	408.0	2.1
Skin diseases	1421.4	604.3	2940.4	2458.6	1015.6	5156.5	73.0
Musculoskeletal diseases	414.0	249.1	658.9	582.0	323.0	1002.5	40.6
Rheumatoid arthritis	153.0	81.8	270.9	184.7	90.3	353.4	20.7
Other musculoskeletal disorders	261.0	142.5	453.5	397.3	201.3	738.0	52.2
Congenital anomalies	14467.8	9046.6	21964.1	14442.6	8439.8	23481.2	−0.2
Neural tube defects	5514.5	2460.4	10948.8	3638.5	1526.6	7535.9	−34.0
Cleft lip and cleft palate	161.9	63.3	358.1	177.3	65.9	403.3	9.5
Down syndrome	241.8	96.2	527.5	219.2	81.9	496.4	−9.4
Congenital heart anomalies	3130.6	1369.5	6321.9	3820.5	1604.7	7905.6	22.0
Other chromosomal anomalies	450.2	183.5	963.7	775.2	307.4	1678.6	72.2
Other congenital anomalies	4968.8	2207.5	9901.3	5811.9	2479.1	11875.3	17.0
Sudden infant death syndrome	1269.7	537.6	2635.3	1107.8	443.5	2381.5	−12.7

NCD: noncommunicable disease.

**Table 2:** Crude and ASDR/100000 population from NCD for sex and all age groups, 2000-2016.

Cause by group	CDR			ASDR		
	2000	2016	%Change	2000	2016	%Change
All causes	1475.8	683.7	−53.7	1816.7	1048.3	−42.3
Noncommunicable diseases	299.5	268.5	−10.3	634.3	554.7	−12.5
Malignant neoplasms	51.9	49.7	−4.2	103.6	93.5	−9.7
Mouth and oropharynx cancers	1.3	1.3	2.5	2.6	2.5	−5.7
Oesophagus cancer	2.2	1.6	−26.2	4.8	3.4	−29.3
Stomach cancer	2.2	1.5	−34.1	4.8	3.0	−37.1
Colon and rectum cancers	3.6	3.4	−4.7	7.4	6.7	−10.1
Liver cancer	1.0	1.1	9.4	2.0	2.1	3.4
Pancreas cancer	0.4	0.5	20.8	1.0	1.2	12.2
Trachea, bronchus, and lung cancers	1.5	1.6	8.7	3.2	3.3	5.2
Melanoma and other skin cancers	0.2	0.2	7.9	0.5	0.5	−1.5
Breast cancer	7.8	7.5	−4.1	14.8	13.4	−9.5
Cervix uteri cancer	6.7	4.9	−26.4	13.9	9.9	−29.1
Corpus uteri cancer	0.3	0.2	−12.8	0.6	0.5	−17.9
Ovary cancer	1.8	2.5	35.9	3.6	4.6	27.5
Prostate cancer	1.1	1.4	28.0	3.0	3.2	7.5
Testicular cancer	0.1	0.1	−39.3	0.2	0.1	−42.9
Kidney cancer	1.1	1.3	12.4	1.6	1.8	16.1
Bladder cancer	0.9	0.9	−1.4	2.2	2.0	−8.9
Brain and nervous system cancers	0.4	0.5	25.2	0.7	0.9	18.6
Gallbladder and biliary tract cancer	0.7	0.6	−14.3	1.3	1.1	−18.4
Larynx cancer	0.2	0.2	−14.7	0.5	0.4	−19.8
Thyroid cancer	1.6	1.4	−13.6	3.6	3.0	−18.5
Mesothelioma	0.1	0.1	−29.4	0.2	0.1	−35.3
Lymphomas, multiple myeloma	2.9	3.0	6.1	5.3	5.4	2.4
Leukaemia	3.5	3.4	−2.6	6.0	5.6	−5.9
Other malignant neoplasms	10.4	10.7	2.2	19.6	18.9	−3.7
Other neoplasms	1.7	2.5	47.3	3.2	4.5	43.2
Diabetes mellitus	12.1	12.7	5.0	29.9	29.1	−2.8
Endocrine, blood, and immune disorders	7.2	4.0	−43.6	7.6	5.5	−27.4
Thalassaemias	1.9	0.7	−61.6	0.9	0.4	−52.2
Sickle cell disorders and trait	1.6	0.7	−54.9	0.9	0.5	−45.7
Other haemoglobinopathies	2.8	1.6	−41.5	4.2	2.8	−33.3
Other endocrine and immune disorders	1.0	1.0	3.4	1.6	1.8	13.8
Mental and substance use disorders	1.9	1.9	0.2	3.5	3.2	−8.9
Alcohol use disorders	1.4	1.3	−11.2	2.8	2.4	−15.3
Drug use disorders	0.4	0.6	37.5	0.7	0.9	15.1
Eating disorders	0.0	0.0	18.1	0.0	0.0	10.5
Neurological conditions	10.9	12.4	13.3	28.4	30.3	6.8
Alzheimer disease and other dementias	5.4	8.1	49.5	21.2	24.5	15.5
Parkinson disease	0.3	0.5	59.6	1.2	1.5	27.5
Epilepsy	4.7	3.3	−30.8	5.5	3.7	−32.0
Multiple sclerosis	0.0	0.0	11.7	0.1	0.1	4.9
Other neurological conditions	0.4	0.4	4.3	0.5	0.5	12.7
Cardiovascular diseases	114.6	106.9	−6.7	292.9	252.9	−13.7
Rheumatic heart disease	3.0	1.4	−53.3	5.5	2.7	−50.9
Hypertensive heart disease	7.7	7.6	−1.3	21.0	18.8	−10.7
Ischemic heart disease	45.2	46.6	3.1	119.5	112.4	−5.9
Stroke	39.9	32.1	−19.5	101.1	74.9	−26.0
Cardiomyopathy, endocarditis	2.9	2.9	0.1	5.3	5.4	3.0
Other circulatory diseases	15.9	16.4	2.7	40.5	38.7	−4.5
Respiratory diseases	18.5	15.1	−18.3	43.0	34.0	−20.9
Chronic obstructive pulmonary disease	11.0	10.1	−8.2	29.5	24.6	−16.8
Asthma	6.8	4.4	−35.8	12.3	8.3	−32.2
Other respiratory diseases	0.7	0.6	−6.9	1.1	1.1	−5.6
Digestive diseases	44.2	36.0	−18.6	86.3	69.5	−19.5
Peptic ulcer disease	6.4	4.1	−35.7	14.2	9.0	−36.9
Cirrhosis of the liver	18.0	16.2	−9.9	35.3	30.9	−12.5
Appendicitis	2.1	1.2	−42.6	2.6	1.7	−34.1
Gastritis and duodenitis	1.1	1.1	0.0	2.5	2.3	−6.8
Paralytic ileus and intestinal obstruction	8.6	7.3	−15.9	15.5	13.2	−14.7
Inflammatory bowel disease	0.7	0.8	7.2	1.3	1.4	6.3
Gallbladder and biliary diseases	1.2	1.2	−0.3	2.8	2.6	−7.3
Pancreatitis	0.7	0.7	−8.8	1.4	1.2	−12.7
Other digestive diseases	5.5	3.6	−34.9	10.8	7.2	−32.9
Genitourinary diseases	10.1	9.1	−9.8	19.7	18.0	−9.0
Kidney diseases	6.8	6.3	−8.2	13.7	12.7	−7.5
Urolithiasis	0.1	0.1	27.5	0.1	0.1	13.3
Other urinary diseases	2.9	2.6	−11.4	5.4	4.8	−10.5
Gynecological diseases	0.3	0.2	−33.7	0.5	0.3	−36.6
Skin diseases	2.1	2.4	12.4	3.7	4.1	10.2
Musculoskeletal diseases	0.6	0.6	−8.7	1.0	0.9	−10.3
Rheumatoid arthritis	0.2	0.2	−21.6	0.5	0.4	−22.3
Other musculoskeletal disorders	0.4	0.4	−1.1	0.5	0.5	0.6
Congenital anomalies	21.7	14.1	−35.1	10.5	8.5	−19.0
Neural tube defects	8.3	3.6	−57.1	4.0	2.1	−46.3
Cleft lip and cleft palate	0.2	0.2	−28.8	0.1	0.1	−10.4
Down syndrome	0.4	0.2	−41.1	0.2	0.1	−26.3
Congenital heart anomalies	4.7	3.7	−20.7	2.3	2.3	−2.2
Other chromosomal anomalies	0.7	0.8	11.9	0.3	0.5	40.0
Other congenital anomalies	7.5	5.7	−24.0	3.6	3.4	−5.2
Sudden infant death syndrome	1.9	1.1	−43.3	0.9	0.6	−28.7

CDR: crude death rate; ASDR: age-standardized death rate; NCD: noncommunicable disease.

**Table 3:** DALY from NCD in thousands for both sexes and all age groups in Ethiopia, 2000-2016.

Causes of DALY	2000	2016	%Change
All causes	71354.0	46507.4	−34.8
Noncommunicable diseases	12053.5	15849.8	31.5
Malignant neoplasms	1386.7	2012.5	45.1
Mouth and oropharynx cancers	35.5	55.3	55.9
Oesophagus cancer	47.8	53.0	11.0
Stomach cancer	52.6	51.6	−1.7
Colon and rectum cancers	88.6	127.8	44.3
Liver cancer	25.1	42.4	68.9
Pancreas cancer	9.6	17.7	83.8
Trachea, bronchus, and lung cancers	35.6	58.6	64.7
Breast cancer	226.9	327.0	44.2
Cervix uteri cancer	163.2	180.3	10.4
Ovary cancer	47.2	97.6	107.0
Prostate cancer	18.2	35.3	94.2
Kidney cancer	43.3	71.3	64.8
Bladder cancer	18.2	26.6	46.3
Brain and nervous system cancers	11.9	22.4	88.5
Gallbladder and biliary tract cancer	17.3	22.8	31.9
Thyroid cancer	29.0	37.5	29.3
Lymphomas, multiple myeloma	84.1	133.4	58.6
Leukaemia	112.1	161.1	43.7
Other malignant neoplasms	298.6	462.6	54.9
Other neoplasms	49.9	104.4	109.3
Diabetes mellitus	346.4	528.7	52.6
Endocrine, blood, and immune disorders	411.8	340.4	−17.3
Thalassaemias	133.1	102.7	−22.8
Sickle cell disorders and trait	94.2	67.3	−28.6
Other haemolytic anaemias	124.3	95.1	−23.5
Other endocrine, blood, and immune disorders	60.2	75.3	25.1
Mental and substance use disorders	1304.8	2089.7	60.1
Alcohol use disorders	161.3	287.5	78.2
Drug use disorders	67.0	124.6	85.8
Eating disorders	7.4	16.0	115.5
Neurological conditions	614.8	895.2	45.6
Alzheimer disease and other dementias	67.6	141.1	108.6
Epilepsy	308.8	354.2	14.7
Other neurological conditions	37.7	63.7	69.2
Cardiovascular diseases	2375.0	3009.9	26.7
Rheumatic heart disease	118.9	97.6	−18.0
Hypertensive heart disease	137.6	184.4	34.0
Ischemic heart disease	844.6	1191.3	41.0
Stroke	787.4	885.7	12.5
Cardiomyopathy, myocarditis, and endocarditis	98.5	128.6	30.6
Other circulatory diseases	388.1	522.4	34.6
Respiratory diseases	605.3	721.1	19.1
Chronic obstructive pulmonary disease	240.9	315.0	30.8
Asthma	337.9	367.7	8.8
Other respiratory diseases	26.5	38.4	44.7
Digestive diseases	1341.3	1483.3	10.6
Peptic ulcer disease	162.0	134.7	−16.9
Cirrhosis of the liver	510.9	651.7	27.6
Appendicitis	89.3	68.7	−23.1
Gastritis and duodenitis	31.1	45.9	47.8
Paralytic ileus and intestinal obstruction	287.7	321.2	11.6
Inflammatory bowel disease	23.3	33.6	44.2
Gallbladder and biliary diseases	26.1	34.9	33.7
Pancreatitis	22.0	28.9	31.3
Other digestive diseases	188.9	163.6	−13.4
Genitourinary diseases	467.5	622.5	33.2
Kidney diseases	260.4	337.3	29.5
Other urinary diseases	92.0	107.9	17.3
Gynecological diseases	86.1	126.6	47.2
Skin diseases	284.8	450.0	58.0
Musculoskeletal diseases	390.4	679.9	74.2
Rheumatoid arthritis	23.1	35.4	53.7
Other musculoskeletal disorders	92.7	159.7	72.3
Congenital anomalies	1444.5	1497.6	3.7
Neural tube defects	529.4	371.9	−29.7
Down syndrome	23.1	21.9	−5.5
Congenital heart anomalies	286.9	350.8	22.2
Other chromosomal anomalies	46.7	79.0	69.1
Other congenital anomalies	542.9	657.0	21.0
Sudden infant death syndrome	115.9	101.3	−12.7

## Data Availability

The data used to support the findings of this study are available from the corresponding author upon request.

## Abbreviations

ASDR: Age-standardized death rate
CODEm: Cause of death ensemble modeling
DALY: Disability-adjusted life years
GBD: Global Burden of Disease
MDGs: Millennium development goals
NCDs: Noncommunicable diseases
WHO: World Health Organization.
